# In vitro assessment of the anthelmintic activity of copper oxide and zinc oxide nanoparticles on egg and adult stages of *Fasciola hepatica*: evidence on oxidative stress biomarkers, and DNA damage

**DOI:** 10.1186/s12917-024-03994-0

**Published:** 2024-04-04

**Authors:** Amirhassan Ravvaz, Farnaz Malekifard, Bijan Esmaeilnejad

**Affiliations:** Department of Pathobiology, Faculty of Veterinary Medicine, Urmia University, P.O. Box 1177, Urmia, Iran

**Keywords:** Copper oxide nanoparticles, DNA damage, *Fasciola hepatica*, In vitro, Anthelmintic activity, Oxidative stress, Zinc oxide nanoparticles

## Abstract

**Objectives:**

Fasciolosis is of significant economic and public health importance worldwide. The lack of a successful vaccine and emerging resistance in flukes to the drug of choice, triclabendazole, has initiated the search for alternative approaches. In recent years, metallic nanoparticles have been extensively investigated for their anthelmintic effects. This study investigates the in vitro anthelmintic activity of copper oxide and zinc oxide nanoparticles against *Fasciola hepatica*.

**Methods:**

The in vitro study was based on egg hatchability test (EHA), adult motility inhibition tests, DNA damage, ROS levels, as well as several biomarkers of oxidative stress, including glutathione peroxidase (GSH) and glutathione S-transferase (GST), superoxide dismutase (SOD) and malondialdehyde (MDA). For this purpose, different concentrations of copper oxide nanoparticles (CuO-NPs) and Zinc oxide nanoparticles (ZnO-NPs) (1, 4, 8, 12, and 16 ppm) were used to evaluate the anthelmintic effect on different life stages, including egg and adults of *Fasciola hepatica*, over 24 h.

**Results:**

In vitro treatment of *F. hepatica* worms with both CuO-NPs and ZnO-NPs could significantly increase ROS production and oxidative stress induction (decreased SOD, GST and GSH and increased MDA) compared to control group.

**Conclusions:**

Based on the results, it seems that CuO-NPs and ZnO-NPs may be effective in the control and treatment of *F. hepatica* infection. Further research is needed to investigate their potential for in vivo use in the treatment of parasitic infections.

**Supplementary Information:**

The online version contains supplementary material available at 10.1186/s12917-024-03994-0.

## Introduction

Fasciolosis is an emerging zoonosis disease caused by the leaf-shaped trematode called *Fasciola hepatica.* Although human cases were rare in the past, they are now increasingly reported on five continents. According to the World Health Organization, at least 2.4 million people are infected in over 70 countries worldwide [1]. *F.hepatica* affects the biliary ducts of animals such as cattle, sheep, and goats, leading to significant economic losses [2].

Currently, the use of anthelmintics is the main method of controlling this disease [3]. Triclabendazole (TCBZ) is the drug of choice for treating infected animals. However, there are concerns about increasing reports of drug resistance to TCBZ and other anthelminticdrugs in flukes, necessitating the search for alternative treatment methods [4].

Nanoparticles are used in various scientific fields such as cancer therapy, drug delivery, and medicine due to their nanoscale size and significant properties [5] They are suitable for a variety of biomedical applications due to their ability to produce reactive oxygen species (ROS) makes them an effective means of eliminating infectious agents [6, 7]. Due to their small size, nanoparticles can easily pass through membrane barriers and cause higher reactivity [8]. Many environmentally friendly and effective nanoparticles have been successfully prepared for the elimination of intestinal parasites [9–13].

Copper is a widely used element in various industries, including electrical, due to its affordability. Recently, copper oxide nanoparticles (CuO-NPs) and other metal nanoparticles have been used to prevent and control parasites such as mosquito larvae and *Giardia duodenalis* due to their powerful effects [14, 15].

Zinc is an essential element for human health but can be toxic to microorganisms [16]. Zinc oxide (ZnO) is a mineral found in zincite that is non-toxic and is often used for skin conditions in humans [17]. Zinc oxide nanoparticles (ZnO-NPs) have received increasing attention due to their safety for humans and animals, as well as their stability under various conditions [11]. These nanoparticles possess various physicochemical properties that make them highly effective as antibacterial and antiparasitic agents [10, 11, 18].

The current study hypothesized that copper oxide and zinc oxide nanoparticles can be used as anthelmintics by inducing DNA damage and oxidative stress due to the ability of metallic nanoparticles to cause oxidative stress and form free radicals inside biological systems [12]. Therefore, this study aims to evaluate the anthelmintic effects of CuO-NPs and ZnO-NPs by measuring various parameters, such as egg hatching and adult worm motility. Furthermore, the study investigates the effect of CuO-NPs and ZnO-NPs on the generation of oxidative stress by measuring several biomarkers of oxidative stress, including ROS, SOD, GSH, MDA, GST, and DNA damage, using in vitro approaches.

## Materials and methods

### Ethics approval and consent to participate

All of the protocols were approved by the Faculty of Veterinary Medicine’s Committee on the Ethics of Animal Experiments at Urmia University (IR-UU-AEC-3/62).

### Nanoparticles (NPs)

Copper oxide nanoparticles (CuO-NPs; stock # us3070; size = 10–40 nm) and zinc oxide nanoparticles (ZnO-NPs; stock # us3590; size = 10–30 nm) were purchased from a commercial supplier (purchased from Iranian Nanomaterials Pioneers Company, NANOSANY; Mashhad, Iran). These nanoparticles were originally manufactured by US Research Nanomaterials, Inc. USA. To obtain a homogeneous suspension, the nanoparticles were dispersed in highly pure water and sonicated at 100 W and 40 kHz for 40 min. The ZnO-NPs were then serially diluted in sterile ultrapure water and additionally sonicated for 40 min. During dilution, magnetic bars were added to the suspensions to prevent particle aggregation or deposition [19].

### CuO-NPs and ZnO-NPs suspension preparation

To prepare the nanoparticle suspensions with different concentrations, previously described procedures were followed [13]. CuO-NPs and ZnO-NPs stock suspensions were prepared in PBS (pH = 7.4). An ultrasonic probe (Branson Sonifier, USA) was used intermittently for 10 min at 30 W to sonicate the solution and prevent agglomeration while achieving uniform dissolution. By diluting the stock solution, different concentrations of CuO-NPs (1, 4, 8, 12, and 16 ppm) and ZnO-NPs (1, 4, 8, 12, and 16 ppm) were prepared in RPMI 1640 (Sigma-Aldrich Chemie GmbH, Germany) medium supplemented with 5% (/v) fetal bovine serum (FBS; Sigma, USA) and 10 mL/L penicillin-streptomycin solution (Sigma, USA) [11] .

### Parasite collection

The adult flukes of *F. hepatica* were collected from the bile duct and gallbladder of cattle slaughtered at the local slaughterhouse in Urmia city, Iran. The flukes were thoroughly washed in Hanks’ balanced salt solution and then incubated separately in RPMI 1640 medium containing different concentrations of CuO-NPs and ZnO-NPs [3]. Only completely intact and actively motile worms were used for the study.

### Collection and extraction of *F. hepatica* eggs

The technique used by Moazeni and Khademolhoseini (2016) [20] was used to extract *F. hepatica* eggs from the gallbladder of cattle naturally infected with *F. hepatica*. The bile was transferred to glass cylinders in an aseptic environment and allowed to solidify for 30 min. The eggs settled at the bottom of the cylinders, and the remaining liquid was removed. The eggs were then washed several times with normal saline and finally stored in a dark glass container with normal saline at 4 °C for later use.

### Egg hatch test

In this study, *F. hepatica* eggs were exposed to different concentrations of CuO-NPs and ZnO-NPs (1, 4, 8, 12, and 16 ppm) for different time periods (24, 48, and 72 h). For each test, a drop of egg-rich sediment containing at least 1,500 eggs was placed in a test tube filled with 10 mL of each NPs. The exact number of eggs was counted using an optical microscope. The tubes were incubated at 37 °C for 24, 48, and 72 h. Afterwards, 9 mL of the upper part of the solution was removed, avoiding the settled eggs. The eggs were then washed and transferred to small plastic containers containing 5 mL of dechlorinated tap water. The containers were incubated at 28 °C. At the same time, one container containing at least 3,000 eggs with no exposure to NPs was also incubated at 28 °C as a control group. After 14 days of incubation, the eggs were streaked on a manually scaled glass slide, covered with a coverslip, and examined under a light microscope. The potential inhibitory effect of CuO-NPs and ZnO-NPs on *F. hepatica* egg hatching was determined by counting at least 1,000 eggs by microscopic inspection in each experiment. The experiment was repeated three times for each concentration [20].

### In vitro treatment of parasites

To study the in vitro effect of NPs on adult *F. hepatica* worms, a total of 10 worms were cultured in triplicate in 5 mL of RPMI medium supplemented with 5% (v/v) fetal bovine serum containing different concentrations of NPs and incubated for 24 h at 37 ± 1 °C. Triclabendazole (TCBZ 20 µg/mL) and PBS were included in the assay as positive and negative controls, respectively. After incubation, the adult *F. hepatica* was thoroughly rinsed with phosphate-buffered saline. Parasites were homogenized in 100 mM Tris-HCl buffer, pH 7.4, centrifuged at 10,000 × *g* for 30 min at 4 °C, and the supernatant was collected and stored at − 80 °C until use [3].

### Observation on parasite mortality and mobility

In this study, parasite mortality and mobility were monitored every 4 h for up to 24 h after incubating them in different NP concentrations. The mobility of control worms (without NPs) was also recorded. Using a dissecting microscope (SMZ1270, Nikon, Tokyo, Japan), at 2x magnification, the number of live and dead worms was counted and recorded separately for each concentration. A 5-point qualitative scale was used to assess parasite mobility [12, 19]. The experiment was repeated three times before the results were presented as a percentage of mortality. The percentage of mortality was calculated for each concentration using the following formula [10]:

$$\eqalign{{\rm{Mortality}}\,{\rm{(\% )}}\,{\rm{ = }}\, & {\rm{(number}}\,{\rm{of}}\,{\rm{dead}}\,{\rm{worms}}\, \cr & {\rm{/}}\,{\rm{total}}\,{\rm{number}}\,{\rm{of}}\,{\rm{worms}}\,{\rm{per}}\,{\rm{test)}} \cr}$$

### Reactive oxygen species estimation

To determine the amount of superoxide anions generated during treatment with CuO-NPs and ZnO-NPs, the method described by Sim Choi et al. (2006) was used [21] The treated and untreated samples were incubated in a 2% nitroblue tetrazolium (NBT) solution at 37 ± 1 °C for 2 h. The resulting formazan crystals were dissolved in DMSO, and the absorbance was measured at 620 nm.

### Glutathione peroxidase assay

To determine GSH activity, we used the GSH detection kit (Ransel, RanDox Co., UK). The measurement method recommended by the manufacturer was followed and the absorption reduction was measured spectrophotometrically (Shimadzu AA-6800; Kyoto, Japan) at 340 nm using a blank sample [22]. The protein content of the supernatant was measured using the Lowry colorimetric method, using bovine serum albumin (BSA) as a standard. The units are classified based on the protein content of the parasite homogenate [19].

### Glutathione-S-transferase assay

The GST assay was carried out according to the method described by Habig et al. (1974) [23]. The assay used 10 mM GSH and 1 mmol CDNB (1-chloro-2,4-dinitrobenzene) as substrates. To start the assay, 50 µL of protein sample was added to 100 mM potassium phosphate buffer (pH 6.5). Enzyme activity was calculated as nmol of CDNB conjugate per minute per milligram of protein using a molar extinction coefficient of 9.6 × 103 M/cm.

### Estimation of superoxide dismutase (SOD) activity

A standard commercial kit (Randox Laboratories Ltd., Crumlin, UK) was used to determine the SOD activity according to the xanthin-xanthine oxidase assay [24]. The activity of SOD was measured at a wavelength of 505 nm using a standard curve.

### Assessment of lipid peroxidation (MDA)

To determine MDA as a biomarker of lipid peroxidation, we used the method described by Buege and Aust [25]. To do this, we mixed one volume of homogenate with two volumes of a stock solution containing 15% v/v trichloroacetic acid, 0.375% v/v thiobarbituric acid, and 0.25 mol/L hydrochloric acid. After the heating and cooling periods, the resulting solution was centrifuged at 1000 rpm for 10 min to obtain a clear solution. We then determined the absorbance at 535 nm and calculated the MDA content using 1.56 × 105 mol/cm as the molar absorption coefficient. The MDA content was recorded in nmol per mg of protein.

### DNA damage assessment

A modified version of the alkaline comet assay [26] was used to assess DNA damage in *F. hepatica*. The non-invasive extrusion method was employed to collect the coelomocytes of the worms after incubation [27]The comets were visually inspected and scored based on the amount of DNA in their tails [28]The images were grouped based on the fluorescence intensity in the comet tail and assigned a score of 0, 1, 2, 3, or 4. Total scores were expressed in arbitrary units ranging from 0 to 400 [12].

### Statistical analysis

Statistical analysis was performed using SPSS software (version 26, Chicago, IL, USA). The homogeneity of variances was tested using the Levene test. To compare the analyzed parameters between control and treatment groups, one-way and two-way ANOVA as well as the Bonferroni post hoc test were used. The Shapiro-Wilk test was performed to check for normality. Data were presented as mean ± SD (standard deviation) and a *p* value less than 0.05 (*p* ≤ 0.05) was considered statistically significant.

## Results

### Physicochemical characterization of CuO and ZnO nanoparticles

The crystalline nature of CuO-NPs was determined by X-ray diffraction (XRD) patterns. The XRD pattern of CuO-NPs was obtained at room temperature with a PANalytical X’Pert ProTM X-ray diffractometer equipped with a nickel filter using Cu Kα radiation (l = 1.54056 A°) as the X-ray source. The transmission electron microscope (TEM) diameter of CuO-NPs was calculated to be 20 nm on average. Furthermore, TEM characterization of ZnO-NPs with an X-ray diffractometer showed that the highest diffraction points could be attributed to the hexagonal phase of ZnO-NPs with a crystallite size of 212 Å. The spherical structure of ZnO-NPs was visualized using a transmission electron microscope, and the diameters of the spherical structures were 20–30 nm.

The crystalline nature of CuO-NPs was determined by X-ray diffraction (XRD) patterns. The XRD pattern of CuO-NPs was obtained at room temperature with a PANalytical The diameter of CuO-NPs determined by transmission electron microscopy (TEM) was calculated to be 20 nm on average. Furthermore, TEM characterization of ZnO-NPs with an X-ray diffractometer showed that the highest diffraction points could be assigned to the hexagonal phase of ZnO-NPs with a crystallite size of 212 Å. The ball-like structure of ZnO-NPs was observed by a transmission electron microscope, and the diameters of the ball-like structures were 20–30 nm.

### Adult worm motility test

Exposure to different concentrations of CuO-NPs and ZnO-NPs (1, 4, 8, 12, and 16 ppm) for 24 h resulted in a significant decrease in the motility of adult worms. The inhibition rate was higher in the treated worms than in the negative controls (Table 1). In this study, 16 ppm of both NPs completely inhibited the motility of adult worms during the first 12 h of observation (Table 1).

**Table 1:** The effect of various concentrations and incubation time of CuO-NPs and ZnO-NPs on the motility of *Fasciola hepatica*

Hours	Control (-)	Triclabendazole (control +)	CuO-NPs					ZnO-NPs				
			1 ppm	4 ppm	8ppm	12ppm	16 ppm	1 ppm	4 ppm	8 ppm	12 ppm	16 ppm
0 h	++++	++++	++++	++++	++++	++++	++++	++++	++++	++++	++++	++++
4 h	++++	++++	++++	++++	++++	++++	+++	++++	++++	++++	+++	+++
8 h	++++	++++	++++	++++	+++	+++	+	++++	++++	+++	++	+
12 h	++++	++	++++	+++	+++	+	-	++++	++++	+++	++	-
16 h	++++	+	+++	+++	+	-	-	++++	+++	++	-	-
20 h	++++	-	+++	++	-	-	-	+++	++	+	-	-
24 h	+++	-	++	-	-	-	-	++	-	-	-	-

- ++++ (high), +++ (moderate), ++ (low), + (very low), - (no motility)

### Adult worm mortality test

In the adult worm mortality test, increasing the concentration of CuO-NPs and ZnO-NPs and the exposure time resulted in the destruction of adult worms. Adult worms exposed to lower concentrations (1 ppm) showed no adverse effects in the first 4 h interval. However, higher concentrations were able to destroy adult worms within 4 h. The study found that the highest concentration (16 ppm) of both NPs caused 100% mortality within the first 12 h of observation. In this study, 100% mortality was observed in the positive controls within 20 h of the start of observation. In contrast, the mortality rate for negative control was approximately 7.87% after 24 h (Table 2).

**Table 2:** The effect of various concentrations and incubation time of CuO-NPs and ZnO-NPs on the mortality of *Fasciola hepatica*

Hours	Control (-)	Triclabendazole (control +)	CuO-NPs					ZnO-NPs					P value
			1 ppm	4 ppm	8 ppm	12 ppm	16 ppm	1 ppm	4 ppm	8 ppm	12 ppm	16 ppm	
0 h	0.0 ± 0.0 ^Ab^	0.0 ± 0.0 ^Ae^	0.0 ± 0.0 ^Ad^	0.0 ± 0.0 ^Af^	0.0 ± 0.0 ^Af^	0.0 ± 0.0 ^Ae^	0.0 ± 0.0 ^Ad^	0.0 ± 0.0 ^Ad^	0.0 ± 0.0 ^Ae^	0.0 ± 0.0 ^Af^	0.0 ± 0.0 ^Ae^	0.0 ± 0.0 ^Ad^	-
4 h	0.0 ± 0.0 ^Db^	6.99 ± 1.09 ^Dd^	0.0 ± 0.0 ^Dd^	0.56 ± 1.34 ^Df^	11.67 ± 1.35 ^Ce^	25.14 ± 1.32 ^Bd^	39.76 ± 1.21 ^Ac^	0.0 ± 0.0 ^Dd^	0.0 ± 0.0 ^De^	10.62 ± 0.65 ^Ce^	21.54 ± 1.45 ^Bd^	38.22 ± 1.25 ^Ac^	*p* < 0.001
8 h	0.0 ± 0.0 ^Eb^	9.85 ± 0.04 ^Dd^	0.0 ± 0.0 ^Ed^	5.06 ± 2.52 ^DEe^	19.53 ± 1.42 ^Cd^	52.43 ± 0.23 ^Bc^	71.43 ± 0.44 ^Ab^	0.0 ± 0.0 ^Ed^	4.63 ± 0.42 ^DEe^	18.62 ± 1.06 ^Cd^	47.56 ± 1.43 ^Bc^	69.34 ± 1.37 ^Ab^	*p* < 0.001
12 h	0.0 ± 0.0 ^Fb^	32.65 ± 1.86 ^Cc^	9.32 ± 0.45 ^Ec^	21.42 ± 1.43 ^Dd^	37.64 ± 0.77 ^Cc^	73.17 ± 1.54 ^Bb^	100.0 ± 0.0 ^Aa^	8.43 ± 1.13 ^Ec^	19.21 ± 1.05 ^Dd^	35.05 ± 1.65 ^Cc^	72.54 ± 1.38 ^Bb^	100.00 ± 0.0 ^Aa^	*p* < 0.001
16 h	4.43 ± 0.43 ^Fab^	69.08 ± 0.76 ^Cb^	13.86 ± 0.14^Ebc^	44.65 ± 1.07 ^Dc^	81.17 ± 1.65 ^Bb^	100.00 ± 0.0 ^Aa^	100.00 ± 0.0 ^Aa^	12.65 ± 0.08^Eb^	41.32 ± 1.24 ^Dc^	79.05 ± 1.45^Bb^	100.00 ± 0.0 ^Aa^	100.00 ± 0.0 ^Aa^	*p* < 0.001
20 h	5.26 ± 0.64 ^Da^	100.00 ± 0.0 ^Aa^	16.32 ± 0.39^Cb^	83.25 ± 1.27 ^Bb^	100.00 ± 0.0 ^Aa^	100.00 ± 0.0 ^Aa^	100.00 ± 0.0 ^Aa^	15.65 ± 1.06^Cb^	80.22 ± 1.63 ^Bb^	98.9 ± 2.10 ^Aa^	100.00 ± 0.0 ^Aa^	100.00 ± 0.0 ^Aa^	*p* < 0.001
24 h	7.87 ± 0.36 ^Ca^	100.00 ± 0.0 ^Aa^	23.5 ± 1.76 ^Ba^	100.00 ± 0.0 ^Aa^	100.00 ± 0.0 ^Aa^	100.00 ± 0.0 ^Aa^	100.00 ± 0.0 ^Aa^	22.05 ± 0.65 ^Ba^	100.00 ± 0.0 ^Aa^	100.00 ± 0.0 ^Aa^	100.00 ± 0.0 ^Aa^	100.00 ± 0.0 ^Aa^	*p* < 0.001
*P* value	*p* < 0.001	*p* < 0.001	*p* < 0.001	*p* < 0.001	*p* < 0.001	*p* < 0.001	*p* < 0.001	*p* < 0.001	*p* < 0.001	*p* < 0.001	*p* < 0.001	*p* < 0.001	

-Different superscripts (a-f) within the same column indicate a significant toxicity effect of each concentration of NPs within different exposure time. Different superscripts (A-I) within the same row indicate a significant toxicity effect of different concentration of NPs during each exposure times

### Egg hatching test

Table 3 shows that CuO-NPs and ZnO-NPs have a significant effect on preventing egg hatching. CuO-NPs showed a higher percentage of inhibition (100%) at 12 and 16 ppm during 24 h observation (Table 3). The study results after 48 h showed that 8, 12 and 16 ppm CuO-NPs and 12 and 16 ppm ZnO-NPs prevented egg hatching by 100% (Table 3). Furthermore, the results showed that 4, 8, 12, and 16 ppm CuO-NPs and 8, 12, and 16 ppm ZnO-NPs prevented egg hatching even 72 h after the experiment (Table 3).

**Table 3:** Inhibitory effect of CuO-NPs and ZnO-NPs on egg hatch tests against *Fasciola hepatica*

Test	Hours	Control (-)	CuO-NPs					ZnO-NPs					*P* value
			1 ppm	4 ppm	8 ppm	12 ppm	16 ppm	1 ppm	4 ppm	8 ppm	12 ppm	16 ppm	
Inhibition of egg hatch (%)	**24 h**	3.14 ± 1.55 ^Ec^	40.34 ± 1.65 ^Dc^	71.45 ± 1.54 ^Cc^	97.65 ± 2.32^Aa^	100.00 ± 0.00^Aa^	100.00 ± 0.00^Aa^	39.45 ± 1.57 ^Dc^	69.54 ± 1.64 ^Cc^	88.14 ± 2.25 ^Bb^	98.04 ± 2.86 ^Aa^	100.00 ± 0.00^Aa^	*p* < 0.001
	**48 h**	10.45 ± 0.43^Db^	53.24 ± 1.06 ^Cb^	88.43 ± 1.51 ^Bb^	100.00 ± 0.00^Aa^	100.00 ± 0.00^Aa^	100.00 ± 0.00^Aa^	50.73 ± 1.21 ^Cb^	84.54 ± 2.32 ^Bb^	99.26 ± 2.65 ^Aa^	100.00 ± 0.00^Aa^	100.00 ± 0.00^Aa^	*p* < 0.001
	**72 h**	23.5 ± 1.23 ^Ca^	78.09 ± 1.35 ^Ba^	100.00 ± 0.00^Aa^	100.00 ± 0.00^Aa^	100.00 ± 0.00^Aa^	100.00 ± 0.00^Aa^	74.65 ± 1.54 ^Ba^	97.54 ± 2.32 ^Aa^	100.00 ± 0.00 ^Aa^	100.00 ± 0.00^Aa^	100.00 ± 0.00^Aa^	*p* < 0.001

- Different superscripts (a-c) within the same row column indicate a significant inhibitory effect of each concentration of NPs within different exposure time. Different superscripts (A-D) within the same row indicate a significant inhibitory effect of different concentration of NPs during each exposure times

### Generation of ROS

To measure ROS generation in the worms, the amount of superoxide anions generated upon treatment with CuO-NPs and ZnO-NPs was measured. The worms treated with 8, 12, and 16 ppm CuO-NPs and ZnO-NPs showed a concentration-dependent increase in cellular ROS production. This was evidenced by increased absorption values ​​compared to control worms (Table 4).

**Table 4:** The effect of various concentrations of CuO-NPs and ZnO-NPs on oxidative stress parameters and DNA damage after 24 h

Test	Control (-)	CuO-NPs					ZnO-NPs					P value
		1 ppm	4 ppm	8 ppm	12ppm	16 ppm	1 ppm	4 ppm	8 ppm	12 ppm	16 ppm	
SOD (U/mg Pro)	1.54 ± 0.52 ^D^	1.97 ± 0.43 ^BC^	2.53 ± 1.32 ^A^	2.11 ± 0.16 ^B^	1.51 ± 0.43 ^D^	1.13 ± 1.21 ^E^	1.85 ± 1.76 ^C^	2.41 ± 0.65 ^B^	2.03 ± 1.81 ^B^	1.48 ± 0.75 ^D^	1.09 ± 1.76 ^E^	*p* < 0.001
ROS (absorbance@620 nm)	1.07 ± 0.05 ^C^	1.13 ± 0.32 ^C^	1.26 ± 1.08 ^C^	1.38 ± 0.32 ^B^	1.68 ± 1.43 ^AB^	1.87 ± 0.52 ^A^	1.11 ± 1.37 ^C^	1.20 ± 0.68 ^E^	1.37 ± 1.53 ^BC^	1.61 ± 0.87 ^B^	1.83 ± 1.44 ^A^	*p* < 0.001
GSH (U/mg Pro)	27.35 ± 0.76 ^A^	26.55 ± 0.32^AB^	23.32 ± 0.16^BC^	20.65 ± 0.32^CD^	17.23 ± 0.49 ^D^	15.34 ± 0.43 ^D^	26.43 ± 0.75^AB^	22.25 ± 1.45^AB^	19.43 ± 0.65^CD^	16.06 ± 1.18 ^D^	15.09 ± 0.12 ^D^	*p* < 0.001
MDA (nmol/mg Pro)	0.39 ± 0.07 ^D^	0.41 ± 1.22 ^D^	0.49 ± 0.84^D^	0.73 ± 1.45 ^C^	0.86 ± 0.32 ^B^	0.97 ± 1.65 ^A^	0.40 ± 0.24 ^D^	0.48 ± 1.67 ^D^	0.71 ± 0.89 ^C^	0.83 ± 1.75 ^B^	0.94 ± 1.09 ^AB^	*p* < 0.001
GST (U/mg Pro)	35.76 ± 1.08 ^A^	34.78 ± 0.36 ^A^	32.4 ± 0.48 ^AB^	29.8 ± 2.76 ^BC^	27.9 ± 1.43 ^C^	23.4 ± 1.76 ^E^	33.76 ± 1.65^AB^	31.09 ± 1.94^BC^	28.4 ± 1.74 ^CD^	25.76 ± 1.32^DE^	22.8 ± 0.22 ^E^	*p* < 0.001
DNA damage (nmol/mg Pro)	4.78 ± 0.65 ^E^	5.23 ± 1.65 ^DE^	8.06 ± 0.65 ^D^	15.5 ± 1.76 ^BC^	18.45 ± 1.47 ^B^	22.87 ± 1.65 ^A^	5.05 ± 1.35 ^D^	7.45 ± 0.76 ^DE^	14.05 ± 1.85 ^C^	17.65 ± 1.13^BC^	21.76 ± 0.45 ^A^	*p* < 0.001

-SOD: Superoxide dismutase; GSH: Glutathione peroxidase; MDA: Malondialdehyde; ROS: Reactive Oxygen Species; GST: Glutathione-S-transferase. Different superscripts (A-H) within the same row indicate a significant effect

### Superoxide dismutase activity

It was found that the activity of SOD, the main antioxidant enzyme of *F. hepatica* worms, was significantly reduced. The higher concentrations of 16 ppm of the CuO-NPs and ZnO-NPs produced a maximum inhibitory effect, while the lowest concentration (4.8 ppm) caused an increase in SOD activity (Table 4).

### Measurement of GSH activity

The concentration of GSH was significantly reduced upon treatment with CuO-NPs and ZnO-NPs. As shown in Table 4, the activity of GSH decreased significantly (*p* ≤ 0.05) after exposure to different concentrations of CuO-NPs and ZnO-NPs.

### Glutathione-S-transferase activity

The specific activity of GST was significantly reduced when the worms were treated with higher concentrations of 12 and 16 ppm CuO-NPs and ZnO-NPs (Table 4).

### Assessment of lipid peroxidation

The content of MDA, a major end product of the lipid peroxidation process under oxidative stress, was found to increase in a concentration-dependent manner. Although there was no significant change in MDA values at the lowest concentration (1 ppm), a significant increase in MDA values was associated with an increase in the concentration of CuO-NPs and ZnO-NPs compared to control worms (Table 4).

### DNA damage

The study examined the extent of DNA damage caused by *F. hepatica* in tail DNA. The results showed that the concentration of CuO-NPs and ZnO-NPs had a significant effect on DNA damage compared to the negative controls. At the highest NPs concentration (16 ppm), damage increased fivefold compared to negative controls.

## Discussion

NPs are effectively used to control a variety of parasitic diseases. However, it must be taken into account that they can also potentially lead to harmful biological effects at the cellular level. Therefore, after establishing non-cytotoxicity and clinical studies, the NPs can find extensive applications as antiparasitic agents among consumers [29]. They target parasite viability, reduce worm burden, inhibit egg production, and alter the levels of antioxidant enzymes in the worms. In addition, they induce apoptosis in the worms [19, 30, 31].

This study investigated the potential inhibitory effect of CuO-NPs and ZnO-NPs on *F. hepatica* eggs hatching. The study found that 4, 8, 12, and 16 ppm CuO-NPs and 8, 12 and 16 ppm ZnO-NPs could inhibit egg hatching. Other studies have also reported the ovicidal effect of NPs against worm eggs. In an in vitro study, Jalali et al. (2021) [19] showed that CuO-NPs and ZnO-NPs had ovicidal effects on *Marshallagia marsalli* eggs. Their study found that 8, 12 and 16 ppm CuO-NPs and 16 ppm ZnO-NPs could inhibit egg hatching. Baghbani et al. (2020) [12] reported the anthelmintic activity of ZnO-NPs on the eggs of *Teladorsagia circumcincta* at different concentrations and times, while Esmaeilnejad et al. (2018) [11] found that ZnO-NPs at the doses used showed high effectiveness in controlling egg hatching in *Haemonchus contortus* eggs.

Worm motility has long been considered an important factor in testing the effectiveness of anthelmintics because worms must move to find suitable microhabitats and obtain food from the host [32]. In the current study, exposure to the highest concentration (16 ppm) of both NPs for 16 h can affect the motility of the adult *F. hepatica*. It is important to note that the inhibition rate depends on the exposure time and NP dose. Similarly, in other studies, exposure to different doses of Ag-NP strongly affected the motility of *Gigantocotyle explanatum* in a time-dependent manner [30]. Similar results were showed in a study conducted by Jalali et al. [19] in which the motility of *M. marshalli* vanished after 12 h of exposure to 16 ppm concentrations of CuO-NPs and ZnO-NPs. Bioengineered silver nanoparticles also demonstrated anthelmintic effects on *H. contortus* at various stages of its life cycle, including eggs and adult parasites [33]. By targeting different stages of worm development, the use of nanoparticles can limit the development of resistance and improve the effectiveness of anthelmintic treatment [34].

Oxidative stress is harmful to worms. It can alter the normal function of important enzymes and proteins, alter cellular macromolecules, and promote cell death [35]. Under normal conditions, ROS levels are usually constant, but factors such as medications, stress, and illness can increase ROS levels. ROS mainly target DNA, proteins, and lipids [36, 37]. A variety of drugs and nanomaterial products have been shown to stimulate ROS production and induce apoptosis [19, 30, 38].

ROS-mediated apoptosis has recently been used as an effective strategy to combat parasitic infections, including helminth parasites [35, 39]. Among the various drug and vaccine targets, the glutathione-dependent detoxification system involving glutathione peroxidase (GSH) and glutathione S-transferase (GST) has emerged as a promising candidate. These key enzymes help conjugate reduced glutathione (GSH) to xenobiotics, increasing water solubility and ultimately facilitating their excretion from the flukes [40, 41]. These molecules could be used to validate the effectiveness of new compounds/drugs, as enzymatic and non-enzymatic molecules of the glutathione family play a key role in the survival of flukes due to their involvement in antioxidant and detoxification processes [3].

Treatment of flukes with CuO-NPs and ZnO-NPs dose-dependently increased ROS levels in the worms while reducing their GSH and GST activity compared to untreated controls. This could affect the worms’ ability to defend themselves against oxidative stress. The NBT calorimetric assay showed that both CuO-NPs and ZnO-NPs stimulated the production of ROS, which is consistent with previous studies on the treatment of liver fluke *Fasciola gigantica* with curcumin and thymoquinone [3].

GSH plays multiple roles in the cellular antioxidant defense mechanism, including maintaining the redox state by scavenging ROS. It was observed that a decrease in GSH levels leads to an imbalance in the redox process within the parasites [42], which also applies to the flukes treated with CuO-NPs and ZnO-NPs in our studies as well as in other reported studies [3]. This ultimately disrupts intracellular redox homeostasis and impairs the worms’ ability to scavenge free radicals and electrophilic xenobiotics. The reduction in GSH activity after exposure to different concentrations of CuO-NPs and ZnO-NPs could be due to the destruction of antioxidant enzymes or the degradation of minerals or vitamins [12]. Studies have also shown that during oxidative stress, GSH-related enzymes consume glutathione to detoxify peroxides caused by excessive ROS production, leading to depletion of its substrate [43].

GST, along with its phase II detoxification function, has been reported to help parasites develop resistance to commonly used anthelmintics by catalyzing the conjugation of reduced glutathione via a sulfhydryl group to electrophilic sites of a range of substrates [3]. This prompted us to investigate the effect of Cuo-NPs and ZnO-NPs on the GST molecule. In contrast to the dose-dependent decrease in GSH levels, we observed a significant decrease in GST activity in worms treated with CuO-NPs and ZnO-NPs, as also reported in the liver fluke *F. gigantica* [3]. The present study showed that the activities of GST and GPX decreased in worms incubated with CuO-NPs and ZnO-NPs. Previous studies have shown that CuO-NPs and ZnO-NPs cause oxidative/nitrosative damage to biomolecules [11, 12, 19].

The study found that treating *F. hepatica* with different concentrations of CuO-NPs and ZnO-NPs had different effects. The use of NPs appeared to have caused oxidative stress in the parasites through the production of ROS. As a result, the flukes increased the activity of antioxidant enzymes such as SOD to scavenge the ROS generated by NPs treatment. The SOD enzyme helps convert O_2_^·−^ to H_2_O_2_ [44], along with other antioxidants that form an effective system against ROS. However, this protective system appeared to be disrupted when the worms were treated with the highest concentrations (12 and 16 ppm) of CuO-NPs and ZnO-NPs. The study found a significant degree of inhibition of SOD activity in *F. hepatica* after treatment with the highest NPs concentration. This inhibition may be due to the saturation of enzymes resulting from the overproduction of hydroxyl ions and ROS, rendering the detoxification mechanism ineffective in *F. hepatica*, as has been reported in other liver flukes *G. explanatum* (Khan et al., 2015).

The assessment of genotoxic influences in living organisms can be supported by the quantitative and qualitative analysis of oxidative DNA damage. Reinecke and Reinecke (2004) [45] suggested using the comet assay as a biomarker for genotoxic influences on invertebrates. The results of our study suggest that DNA damage by *F. hepatica* occurs in a concentration-dependent manner. Our results are consistent with the study by Jalali et al. (2021) [19], which showed that adult *Marshallagia marshalli* exposed to CuO-NPs and ZnO-NPs can result in DNA damage. Furthermore, the studies by Esmaeilnejad et al. (2018) [11] and Baghbani et al. (2020) [12] concluded that ZnO nanoparticles can cause DNA damage in *H. contortus* and *T. circumcincta*.

## Conclusion

In conclusion, CuO-NPs and ZnO-NPs showed promising in vitro fascioliscide efficacy against adults and eggs of fluke. Furthermore, the study suggests that both compounds have anthelmintic effects on *F. hepatica* via oxidative damage to biomolecules. The study highlights that the effects are concentration dependent and higher concentrations of CuO-NPs and ZnO-NPs can impair the antioxidant systems of *F. hepatica* and damage lipids, proteins, and DNA. Therefore, the study suggests that both compounds could be further explored to develop innovative drug formulations for the control of helminth infections. It should be noted that nanoparticles can have harmful biological effects at the cellular level. Therefore, it is important to establish non-cytotoxicity and conduct clinical studies before using them as antiparasitic agents in consumers. However, further studies are required to gain more insight into the functional significance of these compounds and to find out to what extent they influence parasites under in vivo conditions and thus lead to sustainable control of liver fluke infections.

### Electronic supplementary material

Below is the link to the electronic supplementary material.

Supplementary Material 1

## Data Availability

This article contains all the data that were created or evaluated during the research.

## Abbreviations

CuO-NPs: copper oxide nanoparticles
ZnO-NPs: Zinc oxide nanoparticles
EHA: egg hatchability test
GSH: glutathione peroxidase
GST: glutathione S-transferase
SOD: superoxide dismutase
MDA: malondialdehyde
NBT: nitroblue tetrazolium
BSA: bovine serum albumin
