# Supplementary figures and images for: In vitro assessment of the anthelmintic activity of copper oxide and zinc oxide nanoparticles on egg and adult stages of Fasciola hepatica: evidence on oxidative stress biomarkers, and DNA damage
Source: BMC Vet Res. 2024 Apr 4;20:137. doi: 10.1186/s12917-024-03994-0 (PMC10993569; doi:10.1186/s12917-024-03994-0)

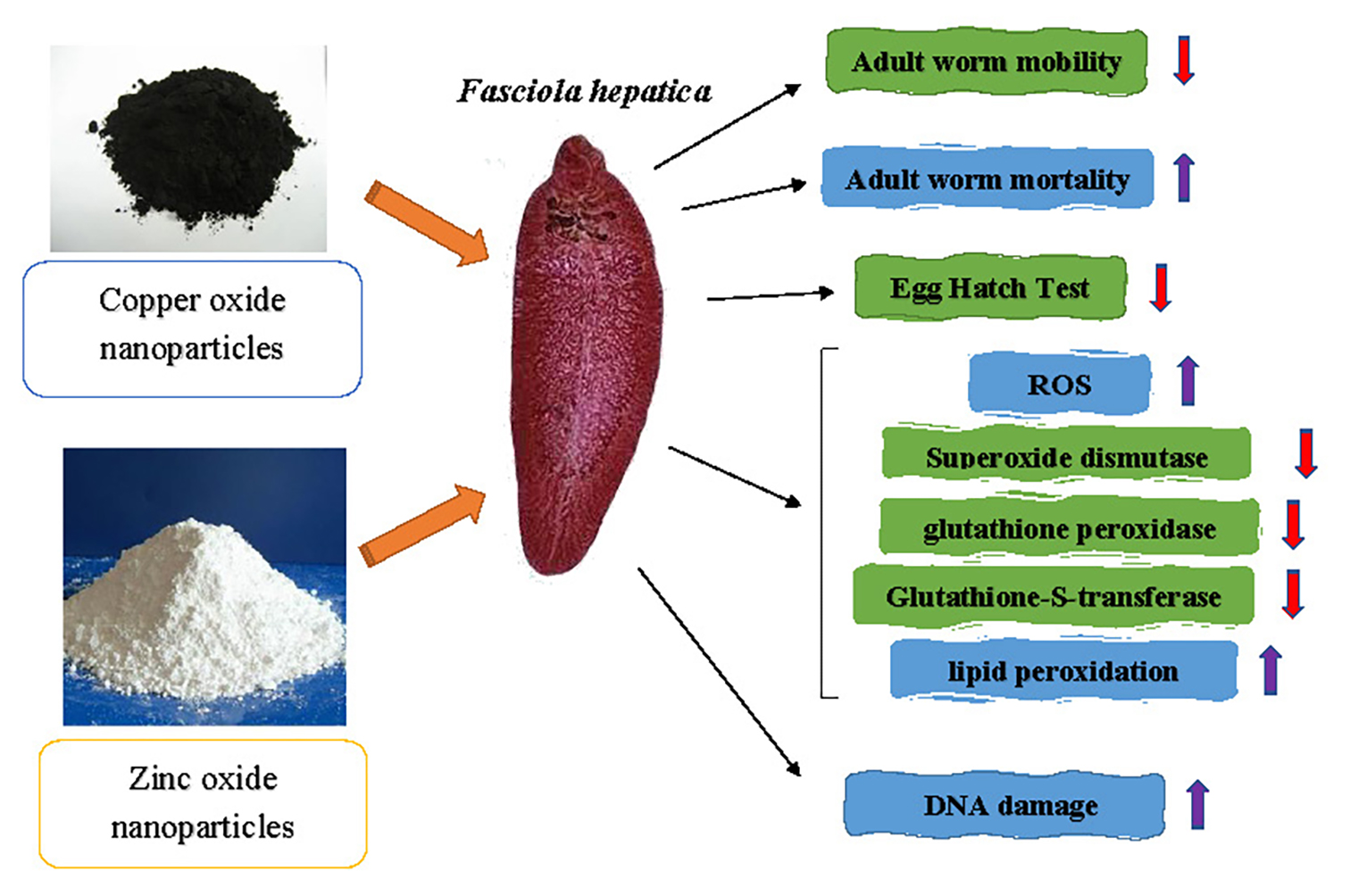

Supplement: Supplementary file 1 — Supplementary Material 1 [file 12917_2024_3994_MOESM1_ESM.bmp]
